# Intracellular Acidification in a Rat C6 Glioma Model following Cariporide Injection Investigated by CEST-MRI

**DOI:** 10.3390/metabo13070823

**Published:** 2023-07-05

**Authors:** Maryam Mozaffari, Nivin N. Nyström, Alex Li, Miranda Bellyou, Timothy J. Scholl, Robert Bartha

**Affiliations:** 1 Department of Medical Biophysics, Schulich School of Medicine and Dentistry, Western University, London, ON N6A 5C1, Canada; scholl@uwo.ca; 2 Centre for Functional and Metabolic Mapping, Robarts Research Institute, Schulich School of Medicine and Dentistry, Western University, London, ON N6A 5B7, Canada; xli328@uwo.ca (A.L.); mbellyou@uwo.ca (M.B.); 3 Department of Chemical Engineering, California Institute of Technology, Pasadena, CA 91125, USA; nnystrom@caltech.edu; 4 Robarts Research Institute, Schulich School of Medicine and Dentistry, Western University, London, ON N6A 5B7, Canada; 5 Ontario Institute for Cancer Research, Toronto, ON M5G 0A3, Canada

**Keywords:** pH, glioblastoma, rat C6 glioma, brain, cancer, chemical exchange saturation transfer, magnetic resonance imaging, cariporide

## Abstract

Acidification of cancerous tissue induced pharmacologically may slow tumor growth and can be detected using magnetic resonance imaging. Numerous studies have shown that pharmacologically inhibiting specific transporters, such as the Na^+^/H^+^ exchanger 1 (NHE1), can alter glycolitic metabolism and affect tumor acidosis. The sodium proton exchanger inhibitor Cariporide can acidify U87MG gliomas in mice. This study aimed to determine whether Cariporide could acidify C6 glioma tumors in rats with an intact immune system. C6 glioma cells were implanted in the right brain hemisphere of ten rats. Chemical exchange saturation transfer (CEST) MRI (9.4T) was acquired on days 7–8 and 14–15 after implantation to measure in vivo tissue intracellular pH (pH_*i*_) within the tumors and on the contralateral side. pH_*i*_ was basic relative to contralateral tissue at both time points assessed using the amine and amide concentration-independent detection (AACID) value. On day 14–15, measurements were made before and up to 160 min after Cariporide injection (N = 6). Twenty minutes after drug injection, the average AACID value in the tumor significantly increased by ∼6.4% compared to pre-injection, corresponding to 0.31 ± 0.20 lower pH_*i*_, while in contralateral tissue, AACID value increased significantly by ∼4.3% compared to pre-injection, corresponding to 0.22 ± 0.19 lower pH_*i*_. Control rats without tumors showed no changes following injection of Cariporide dissolved in 10% or 1% DMSO and diluted in PBS. This study demonstrates the sensitivity of CEST-based pH-weighted imaging for monitoring the response of tumors to pharmacologically induced acidification.

## 1. Introduction

Gliomas are derived from glial cells and are the most common central nervous system (CNS) tumors. Based on the classification of CNS tumors in 2016 by the World Health Organization (WHO) [1], glioblastoma multiform (GBM) is the most frequent astrocytic glioma that arises either de novo or progresses from lower-grade gliomas like diffuse astrocytomas or anaplastic astrocytomas (AA). GBMs have been classified as grade IV gliomas and are more proliferative and infiltrative than lower-grade cancers. Additionally, GBMs are distinguished by the presence of necrotic tissue and glomeruloid microvascular proliferation. Despite the use of multimodal therapies, including surgery followed by concurrent radiotherapy and chemotherapy, and depending on the isocitrate dehydrogenase (IDH) status, the median survival time is approximately 14 months, and treatment remains mainly palliative [1,2,3]. However, there is considerable variability among patients. Prognostic indicators include age, Karnofsky Performance Scale (KPS) score, and the extent of surgical resection [4]. Although there are more than 120 different types of brain cancers, aggressive cancers share similarities in the tissue microenvironment, including altered tissue pH [4].

Cellular pH is tightly controlled in multicellular organisms, and this regulation is required to maintain homeostatic cell growth. The activity of several pH regulators and pumps associated with normal cellular metabolic processes maintain the pH of normal cells within a well-defined physiological range. In tumors, the dysregulation of hydrogen ion dynamics can alter the acidity in cellular compartments and cause a reversal of the normal intracellular/extracellular pH gradient. This reversed pH gradient results from the extrusion of H^+^ from the cytoplasm [5]. The ensuing increase in intracellular pH (pH_*i*_) and decrease in extracellular pH (pH_*e*_) leads to cell transformations such as cell cycle progression, enhanced proliferation, and resistance to apoptosis and angiogenesis [6,7,8,9,10]. Regardless of the genetic background and pathology of the cancer cells, intracellular alkalinization and a shift to glycolytic metabolism are two phenotypes that are common in all aggressive cancers [6,7,8,9,10,11]. The reversed pH gradient in aggressive cancers across the cell membrane occurs due to the activity of a wide variety of acid extrusion mechanisms such as Na^+^-driven extruders, Na^+^/H^+^ exchangers (NHE), V-type H^+^-ATPases and monocarboxylate transporters [12]. Tumor cells generally have an alkaline intracellular pH of 7.1–7.7 compared to 7.0–7.1 in normal cells and an acidic extracellular pH in the 6.2–6.9 range compared to 7.3–7.4 in normal cells [5,13].

The Na^+^/H^+^ exchanger is an integral membrane secondary active acid extruder. The NHE is a ubiquitously expressed plasma membrane protein that exchanges Na^+^ for H^+^ to regulate pH homeostasis. The Na^+^/H^+^ exchanger1 (NHE1) protein from the NHE family is present at the cell surface of most cells and is the main isoform of NHE that has been directly associated with the maintenance of cellular pH, cellular transformation, invasion, and metastasis [13,14,15]. Upregulation of NHE1 expression is correlated with tumor malignancy, and the inhibition of NHE1 reduces tumor cell invasion and motility [16,17,18]. As NHE1 activity elevates, pH_*i*_ increases and pH_*e*_ decreases [12,19]. Therefore, pharmacologic inhibition of NHE1 to induce intracellular acidification is a potential anticancer strategy.

Cariporide (HOE-642) is an NHE1 inhibitor [8,20] that has been clinically tested in the field of ischaemic heart disease and can modify tumor pH_*i*_ [10,13,21]. Previous studies in spontaneous breast cancer and lymphomas in dogs and cats have examined the effects of Cariporide [22]. The chemosensitizing effects of Cariporide have also been evaluated in human malignant mesothelioma cells [23]. Tumor intracellular acidification caused by a single dose of Cariporide has previously been shown in an immunodeficient mouse U87MG glioma model [24]. However, the ability of this drug to induce tumor intracellular acidification in other glioma models, particularly those with an intact immune system, is unknown.

The C6 cell line was initially induced by repetitive intravenous injection of *N*-Nitroso-N-methylurea in adult Wistar-Furth rats [25] and is more invasive than the U87MG model [26,27]. Histologically, the C6 cell line has been classified as an astrocytoma. This glioma cell line overexpresses several genes similar to those reported for human gliomas, such as the PDGFβ, insulin-like growth factor (IGF)-1, epidermal growth factor receptor (EGFR), and Erb3/Her3 precursor proteins [4,28]. Increased activity of the Ras pathway genes has been observed in both human gliomas and C6 cells [29]. Overexpression of CD9 is also found in high-grade glioma, including the C6 glioma cell line [30]. In vivo magnetic resonance imaging (MRI) and angiography have shown that C6 gliomas are morphologically similar to human gliomas [31]. Perhaps most importantly, because the C6 line is derived from rats, it can be implanted into rats with an intact immune system, which is substantially different from U87MG cells implanted in NU/NU mice. A previous study also reported differences in treatment response between the U87MG and rat C6 rat glioma models [32].

Evaluation of drugs designed to modulate intracellular pH must occur in vivo to accurately incorporate the complex response of the tumor microenvironment. Although phosphorus magnetic resonance spectroscopy (^31^P-MRS) is the gold standard for in vivo pH_*i*_ measurement and is feasible in patients with glioma [33,34], the signal-to-noise ratio (SNR) is very low. Consequently, scan times are long and spatial resolution is coarse. Alternatively, changes in tumor pH_*i*_ can be monitored in vivo by chemical exchange saturation transfer (CEST) MRI [35,36]. The CEST mechanism amplifies signals from low-concentration endogenous or exogenous molecules by using selective radio frequency (RF) saturation pulses applied at the resonance frequency of the exchangeable protons in these molecules. Due to chemical exchange processes, magnetization information can transfer from these exchangeable protons to bulk water, reducing the water signal and creating CEST contrast within images [35,37,38]. CEST sensitivity and specificity also increase as a function of magnetic field strength [35]. The magnitude of the CEST contrast is related to the proton exchange rate, which is pH and temperature-dependent [39,40,41]. Several variations of the CEST technique have been applied in clinical oncology research, including the study of brain tumors [42,43,44]. For example, amide proton transfer (APT) imaging contrast has been shown to correlate with molecular subtypes, in particular, the tumor IDH status [45,46,47]. Extracellular pH was also evaluated by CEST following the use of proton pump inhibitors, including Cariporide, in an animal model of prostate cancer [48]. Additionally, CEST contrast changes consistent with a decrease in intracellular tumor pH_*i*_ after injection of a single dose of lonidamine [49], topiramate [50], dichloroacetate [51], and Cariporide [24] have been previously found in a mouse U87MG brain tumor model. Tumor acidification has also been studied in human patients by CEST imaging [44,52], and pH modification has been attempted in some cancers in humans already [53].

Pharmacologically inhibiting specific transporters, such as the NHEs, to disrupt tumor function and/or alter glycolytic metabolism could further enhance GBM cell death. Understanding how the intracellular tumor pH changes after injecting a single dose of Cariporide could help elucidate opportunities for new therapeutic approaches. This study aimed to determine if Cariporide injection caused acidification in C6 glioma tissue in rats with an intact immune system. We hypothesized that blockage of NHE1 by a single dose of Cariporide would acidify C6 rat tumors to the same extent as previously observed in U87MG mouse tumors.

## 2. Materials and Methods

### 2.1. Tissue Culture and Cell Preparation for Implantation

C6 CCL-107 rat glioma cells were acquired from the American Type Culture Collection (ATCC, Manassas, VA, USA). Cells were cultured in 10 cm-diameter tissue culture-treated dishes (08-772-22, Fisher Scientific, Hampton, NH, USA) using Ham’s F12 media (318-010-CL, Wisent Inc., Saint-Jean-Baptiste, QC, Canada) supplemented with 10% fetal bovine serum (FBS, 920-040, Wisent Inc., Saint-Jean-Baptiste, QC, Canada) at 37 °C and 5% CO_2_. Cells were passaged every 3 to 4 days via Trypsin-EDTA (325-043-EL, Wisent Inc., Saint-Jean-Baptiste, QC, Canada). They tested every 3 to 4 months for mycoplasma using the MycoAlert Detection Kit (LT07, Lonza Group, Basel, Switzerland) on a GloMax 20/20 Luminometer (Promega, Madison, WI, USA).

Before implantation, cells were trypsinized, collected, and washed once in Dulbeccos’s phosphate buffered saline (D-PBS, 311-425-CL, Wisent Inc., Saint-Jean-Baptiste, QC, Canada) to remove all cell culture media components before brain injection. A 10 μL-sample was then used for counting and was tested for viability using a 0.4% Trypan Blue solution (15250061, Thermo Fisher Scientific, Hampton, NH, USA). After obtaining the live cell count and confirming high cell viability (>96%), cells were centrifuged a final time, and the pellet was reconstituted in 0.9% sterile saline solution at a concentration of 10^6^ cells per ml for a 10 μL-injection of a million cells.

### 2.2. Chemicals

Cariporide with the chemical formula of C_12_H_17_N_3_O_3_S was purchased from Cayman Chemical Company (Ann Arbor, MI, USA). Cariporide was dissolved in dimethyl sulfoxide (DMSO, Sigma-Aldrich, St. Louis, MO, USA), an organosulfur compound with the formula (CH_3_)_2_SO, and diluted with 1% sterile phosphate buffered saline (PBS, Thermo Fisher Scientific/gibco, Grand Island, NY, USA), a buffer solution with the formula Cl_2_H_3_K_2_Na_3_O_8_P_2_ and pH of 7.4.

### 2.3. Animal Model/Preparation

Male Wistar rats were obtained from Charles River Laboratories (Senneville, QC, Canada). They were cared for under the Canadian Council on Animal Care standards, and experiments were performed according to a protocol approved by Western University’s Council on Animal Care. Tumor cell implantation was initiated when rats were eight weeks old and approximately 250 g. Rats were anesthetized by inhalation of 4% isoflurane in 1.5–2 L/min of oxygen. Rats were placed in a stereotactic head frame (Stoelting Instruments, Wood Dale, IL, USA), and anesthesia was maintained at 2.0% isoflurane in 1.5–2.0 L/min oxygen throughout the surgery. After aseptic preparation of the surgical area, a midline incision was made over the sagittal suture from lambda to slightly past bregma. The periosteum tissue was retracted to create a 2×2 mm^2^ surgical window. A 1 mm-diameter hole was drilled at 3 mm lateral, 1 mm anterior, and 4 mm dorsal-ventral to bregma (at a depth of 1 mm or until dura mater was reached). A 10 μL-suspension containing 10^6^ C6 glioma cells was manually injected at a rate of 3 μL/min at a depth of 3 mm from bregma into the right frontal lobe using a Hamilton syringe (Reno, NV, USA) with a 27-gauge needle attached. The drilled hole was filled with bone wax, and the scalp was sutured. The animal was provided with 0.3 mL of the pain medication Carprofen (Zoetis, Parsippany, NJ, USA) by subcutaneous (SQ) injection (Dose: 5 mg/kg) before the skin incision and for 48 h after the surgery.

### 2.4. Study Overview

**Figure 1:**
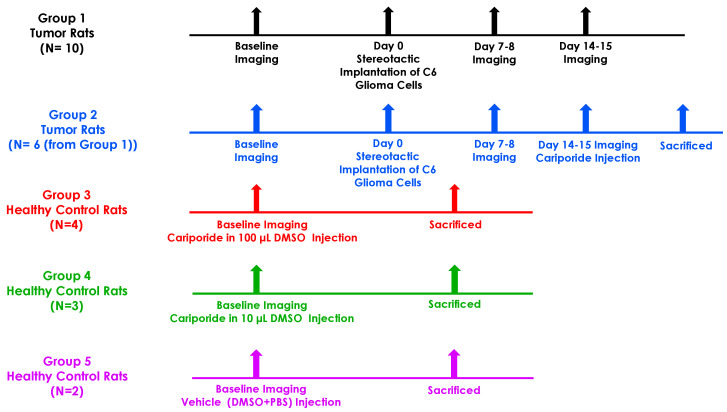
The experimental timeline. Baseline imaging occurred before stereotactic implantation of C6 glioma cells. For group 1, CEST-MRI was performed on day 7–8 and day 14–15 to monitor tumor growth. For group 2, CEST-MRI followed by Cariporide administration was performed on day 14–15. For healthy control groups, groups 3, 4, and 5, CEST-MRI followed by administration of Cariporide in 100 μL DMSO, Cariporide in 10 μL DMSO, and vehicle (DMSO + PBS), respectively, was performed. All animals were sacrificed immediately after imaging was completed.

Twenty-one rats in five groups were included in the current study (Figure 1). One rat died before the first imaging time-point due to neurological impairment. One rat did not grow a tumor, so it was excluded from the study. MRI was performed on ten rats (group 1) at baseline, then 7–8 days, and 14–15 days after tumor implantation. To evaluate the effect of Cariporide on the tumor pH_*i*_, two weeks after tumor implantation, six of those tumor-bearing rats received Cariporide inside the scanner (group 2) (Figure 1). A small number of rats was used due to the large expected effect size. The drug was administrated intraperitoneally (IP) for 30 s at a dose of 6 mg/kg in 2 mL using a Harvard Apparatus (PHD 2000) syringe pump (Harvard Apparatus, Saint-Laurent, QC, Canada) through a non-DEHP micro-volume extension set with a length of 152 cm and volume of 0.4 mL (Baxter Healthcare Corporation, Deerfield, IL, USA) ended with a male Luer lock adaptor and a needle. The effect of Cariporide (dose of 6 mg/kg in 2 mL) dissolved in two different concentrations of DMSO was also studied in healthy control animals. Four healthy control rats (group 3) received Cariporide dissolved in 100 μL of DMSO, the same concentration as those with tumors. Three healthy control rats (group 4) received Cariporide dissolved in 10 μL of DMSO. Additionally, two healthy control rats (group 5) received only vehicle injection (DMSO + PBS). The rats from group 5 were scanned after injection of 2 mL of PBS and then were scanned again after injection of 100 μL of DMSO diluted in 1900 μL of PBS. Animals in all five groups were sacrificed immediately after imaging was completed.

### 2.5. In Vivo Imaging

All imaging was performed at the Centre for Functional and Metabolic Mapping (CFMM) at Western University. MRI data collection was performed on a 9.4T/31cm small animal MRI scanner (Agilent, Palo Alto, CA, USA) equipped with an in-house designed and built 15 cm gradient coil set capable of a maximum gradient strength of 450 mT/m and a Bruker Avance MRI III console running Paravision-6 software (Bruker BioSpin Corp, Billerica, MA, USA). A 6-channel receive-only phased array coil [54] was used in combination with a 2-channel transmit coil for data acquisition. The gradient coil had a full set of second-order shims and five third-order shims. All animal experiments complied with the ARRIVE guidelines. For scanning, anesthesia was induced using 4% isoflurane in 1.5–2 L/min of oxygen and maintained with 1.5–2.0% isoflurane in 1.5–2 L/min oxygen through a custom-built nose cone. The rat was placed on a custom-built stage, and the head was secured using a bite bar and ear bars to limit respiratory motion. Throughout imaging, heart rate, blood-oxygenation-level saturation, respiratory rate, and body temperature were monitored using an MRI-compatible small animal monitoring and gating system (Model 1025 with some upgrades - Small Animal Instrumentals Inc., Stony Brook, NY, USA). The warm air feedback system maintained the animal’s core body temperature at 37.0–37.5 °C.

Each animal was initially imaged when placed in the holder to assess positioning and geometric distortions, and its position was adjusted if needed. B_0_ shimming was performed over an elliptical volume surrounding the brain. A 2-dimensional spin echo (TurboRARE) pulse sequence was used to acquire axial T_2_-weighted images with parameters: TR/TE = 6000/40 ms, FOV = 38.4 × 38.4 mm^2^, matrix size = 192 × 192, 31 slices with thickness = 1 mm, and in-plane resolution = 0.2 mm. After tumor detection, two slices from the T_2_-weighted images with maximum tumor coverage (2 mm thickness) were selected for CEST imaging. CEST images were acquired for the slice of interest using the same 2-dimensional TurboRARE pulse sequence with parameters: TR/TE = 7000/7 ms, FOV = 38.4 × 38.4 mm^2^, matrix size = 96 × 96, slice thickness = 2 mm, in-plane resolution = 0.4 mm, preceded by a continuous wave RF saturation pulse with an amplitude of 1.5 μT and 4s duration. CEST images were acquired at saturation frequencies from 1.2 ppm to 6.6 ppm with a step size of 0.1 ppm. Additional acquisitions at −1000 ppm and +1000 ppm served as reference images. For B_0_ correction, the WAter Saturation Shift Referencing (WASSR) technique was used [55]. Linearly spaced 41-point WASSR CEST spectra were acquired with saturation frequencies ranging from −0.6 ppm to +0.6 ppm with a step size of 0.03 ppm using the same 2-dimensional TurboRARE pulse sequence except preceded by a 100 ms RF saturation pulse with 0.2 μT amplitude. Each WASSR and CEST spectrum (Z-spectrum) was interpolated to a 1-Hz resolution. A smoothing spline generated a Z-spectrum at each pixel using all 57 CEST images. The CEST spectrum for each pixel was then frequency-shifted, using the corresponding WASSR spectrum, to account for regional B_0_ variation. B_0_ variations were corrected on a pixel-by-pixel basis. Three CEST and WASSR images were acquired at the baseline, day 7–8, and day 14–15, then averaged to improve the signal-to-noise ratio (SNR). A B_1_ field map was generated using actual flip-angle imaging (AFI) pulse sequence with parameters: TR = 20/100 ms, TE = 3.47 ms, echoes = 2, flip angle = 70°, FOV = 38.4 × 38.4 mm^2^, matrix size = 64 × 64. The B_1_ variation in the CEST slice was less than 5%, and no B_1_ correction was necessary. Animals received the drug (Cariporide) or vehicle (PBS + DMSO) inside the scanner. Immediately after the injection, CEST images were repeatedly acquired every 20 min up to 160 min.

### 2.6. Image Analysis

A CEST-MRI technique called amine and amide concentration-independent detection (AACID) was previously developed as an indicator of tissue pH_*i*_ [56], and has been applied to measure tumor pH_*i*_ and changes in pH_*i*_ following drug injection [24,49,50,51]. AACID utilizes the ratio of endogenous amine (resonating at M_*z*_ (2.75 ppm)) and amide (resonating at M_*z*_ (3.5 ppm)) CEST effects to measure pH. AACID was developed exploiting the change in the exchange rate of amide and amine protons caused by changes in pH_*i*_. The bulk water magnetization following saturation at M_*z*_ (6 ppm) is a reference point for the amine and amide proton CEST effects. AACID values are typically measured on a pixel-by-pixel basis to generate parametric maps. The AACID value for each pixel was calculated from the B_0_ corrected CEST spectrum using Equation (1) [56]. The AACID value is inversely related to the tissue pH_*i*_, where higher AACID values are associated with lower pH_*i*_. Exchangeable protons are commonly found in amide and amine groups on mobile proteins and peptides predominantly within the intracellular space. Therefore, the measurement is heavily weighted toward the intracellular compartment.

(1) $$\begin{array}{c} \mathrm{AACID}\;=\;\frac{M_{z}\left(3.5\;\mathrm{ppm}\right)\times \left(M_{z}\left(6.0\;\mathrm{ppm}\right)-M_{z}\left(2.75\;\mathrm{ppm}\right)\right)}{M_{z}\left(2.75\;\mathrm{ppm}\right)\times \left(M_{z}\left(6.00\;\mathrm{ppm}\right)-M_{z}\left(3.5\;\mathrm{ppm}\right)\right)} \end{array}$$

All CEST analyses were performed using custom software in MATLAB (Mathworks, Natick, MA, USA) to generate parametric maps. AACID values were measured pixel-by-pixel within manually segmented regions of interest (ROIs). Hyperintense regions on the anatomical T_2_-weighted images were identified as tumors. Tumor, peritumoral, and contralateral ROIs were manually defined (M.M.) using the T_2_-weighted anatomical images. In each rat brain, average AACID values were calculated using MATLAB’s “roipoly” function on days 0, 7–8, and 14–15. AACID values were also calculated after the injection of Cariporide and vehicle. Following drug administration, the change in pH was estimated using Equation (2) provided by McVicar et al. [56], which was obtained from a mouse brain.

(2) $${\mathrm{pH}}_{i}=\;-4\;\times \;\mathrm{AACID}\;+\;12.8$$

T_2_-weighted images were used to manually define tumor tissue to measure volume over time. Tumor volume was measured at day 7–8 and day 14–15 using ITKsnap (www.itksnap.org, accessed on 1 January 2023) [57].

### 2.7. Statistical Analysis

All statistical analyses were performed using GraphPad Prism Version 8.0 for Mac OS X (GraphPad Software, San Diego, CA, USA). Outliers analysis was performed using MATLAB (Mathworks, Natick, MA, USA). Since the study involved a small sample size, a Shapiro-Wilk test was used to determine whether the AACID values were normally distributed. Differences in AACID values between tumor and contralateral regions on day 0, day 7–8, and day 14–15 were identified using two-way repeated measures ANOVA. The side of the brain (tumor versus contralateral), time, and side of the brain/time interaction were considered fixed effects. Sphericity was not assumed, so the Geisser-Greenhouse correction was applied [58]. Similarly, two-way repeated measures ANOVA was used to measure changes in AACID value following injection of Cariporide. When the effect of time was significant, post-hoc Tukey tests were performed to investigate individual differences between time points. In all post-hoc tests, multiplicity-adjusted *p*-values were computed. A paired *t*-test was used to find the differences in tumor volume between day 7–8 and day 14–15. Significance is reported as follows: * = p<0.05, ** = p<0.01, *** = p<0.001, **** = p<0.0001. The number of animals included in each comparison is described in each figure.

## 3. Results

**Figure 2:**
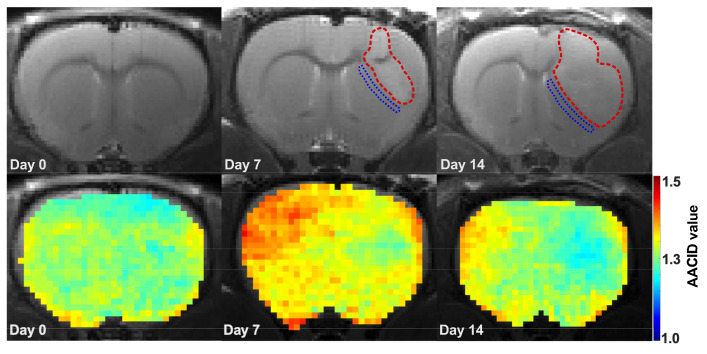
Representative T_2_-weighted anatomical images (top row) and corresponding AACID maps (bottom row) at baseline, Day 7, and Day 14 in one rat. The red and blue lines highlight the tumor and peritumoral regions, respectively.

MR imaging was successfully performed for all animals included in this study. The Shapiro-Wilk test was not significant (W ∼0.9, p>0.01), indicating that the data did not significantly deviate from the normal distribution. Representative anatomical T_2_-weighted images (Figure 2 top row) along with corresponding representative AACID maps (Figure 2 bottom row) for day 0, day 7 and day 14 illustrate the change in tumor size over time in one animal. Tumors are contoured in red. In all animals, the size of tumors increased steadily until the end of the study, with a significant difference in volume between the two imaging days based on a paired *t*-test (p<0.05).

**Figure 3:**
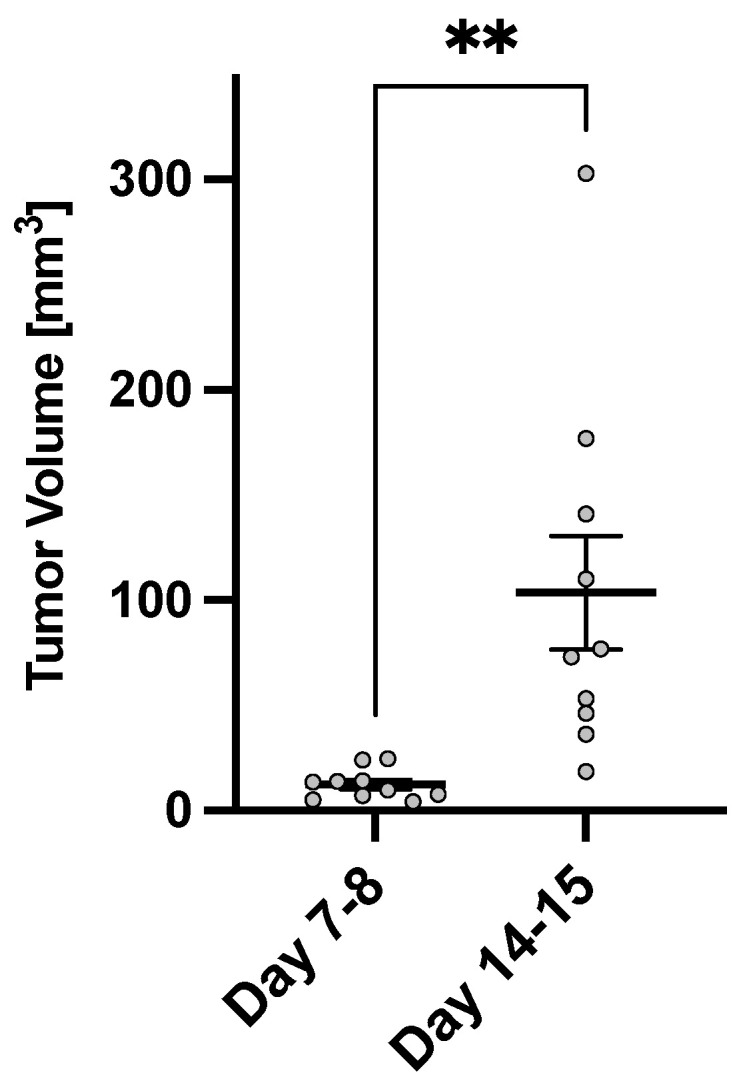
Longitudinal tumor volume measurements (N = 10). The error bars represent the standard error of the mean. Each point represents a measurement from one animal. The asterisk (** = p<0.01) represents statistical significance in the paired *t*-test.

As shown in Figure 3, the average tumor volume 7–8 days post-tumor implantation was 12.4 ± 2.3 mm^3^, while the average tumor volume at 14–15 days post tumor implantation increased to 103.5 ± 27.0 mm^3^.

**Figure 4:**
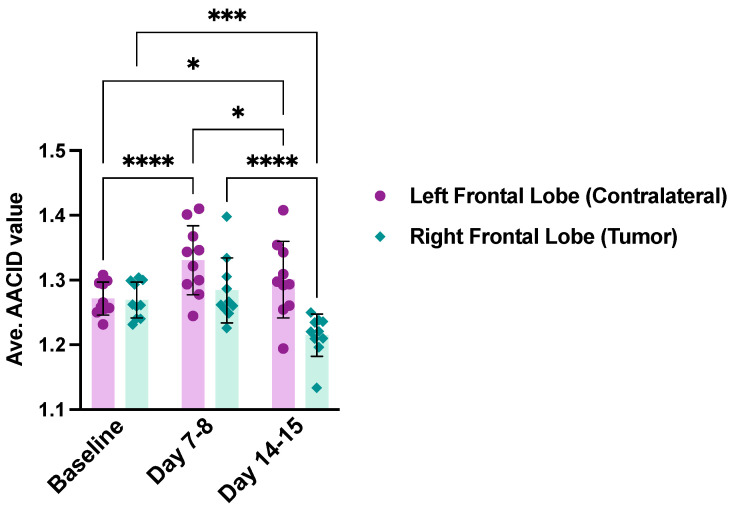
The average AACID values in the right and left frontal lobes at baseline (N = 10), 7–8 days (N = 10), and 14–15 days (N = 10) after tumor implantation. Error bars represent the standard error of the mean. Each point represents a measurement from one animal. The asterisks (* = p<0.05, *** = p<0.001, **** = p<0.0001) denote significant differences in post-hoc Tukey tests.

The AACID maps generated by CEST tracked pH within the rat brain and tumor as the tumor grew. AACID maps obtained for the same rat corresponding to each time point (Figure 2 bottom row) show the pH-related changes over time. The baseline AACID map shows a relatively homogeneous AACID value throughout the brain. As expected, a lower AACID value was observed in the tumor on days 7–8 and 14–15, indicating a small elevation of intracellular tumor pH_*i*_ (basic microenvironment) relative to the contralateral tissue (Figure 2 and Figure 4).

The average AACID values for ten animals at baseline in the right and left frontal lobes and the same animals at 7–8 days and 14–15 days post-tumor implantation are shown in Figure 4. There was no difference in AACID value when comparing the left and right frontal lobes in animals at baseline. On day 7–8, the average AACID value was ∼3.5% lower in the tumor compared to the contralateral side, indicating a 0.18 ± 0.09 higher pH_*i*_ (p<0.001). On day 14–15, the average AACID value was ∼6.6% lower in tumor indicating a 0.34 ± 0.27 higher pH_*i*_ (p<0.0001). When examining changes over time (Figure 4), the contralateral side showed a significant increase in AACID value at day 7–8 followed by a decline from day 7–8 to day 14–15. However, interestingly, the AACID value on day 14–15 was higher than at baseline. Figure 4 shows that within the tumor, the AACID value at day 14–15 was significantly lower (p<0.05) than that observed at both baseline and day 7–8.

**Figure 5:**
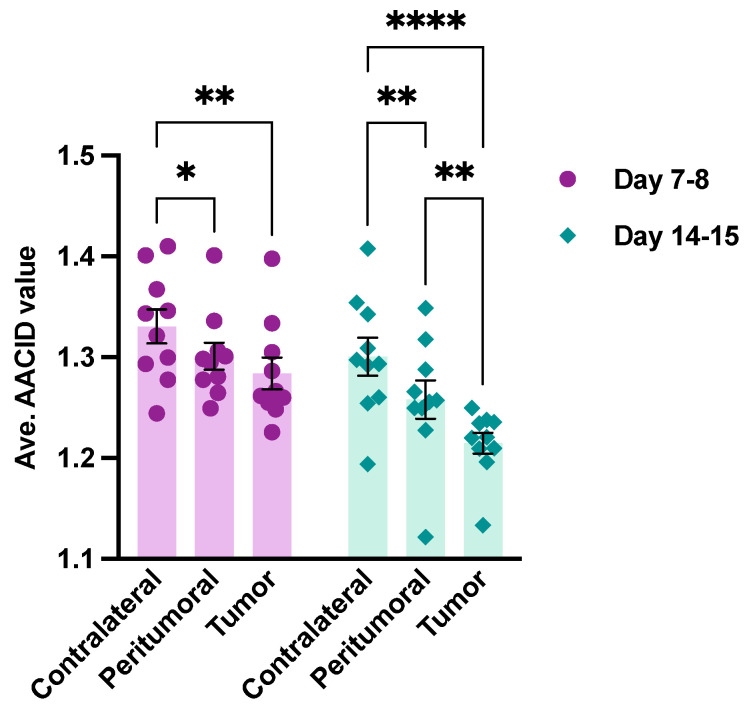
The average AACID values in contralateral, peritumoral, and tumor tissues for the animals at 7–8 days (N = 10) and 14–15 days (N = 10) post-tumor implantation. Error bars denote the standard error of the mean. Each point represents a measurement from one animal. The asterisks (* = p<0.05, ** = p<0.01, **** = p<0.0001) show significant differences in post-hoc Tukey tests.

The average AACID values in contralateral, peritumoral, and tumor tissues at 7–8 days (N = 10) and 14–15 days (N = 10) post-tumor implantation are shown in Figure 5. After comparing the AACID values in peritumoral and tumor tissues, no significant decrease was observed 7–8 days after tumor implantation, whereas a significant decrease was found 14–15 days post-implantation.

**Figure 6:**
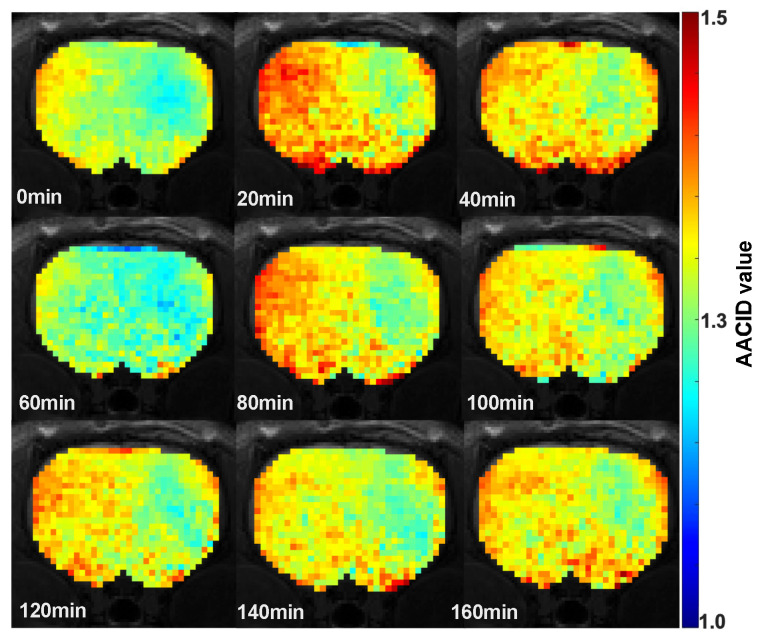
The quantitative AACID distribution maps obtained for a representative animal superimposed on an anatomical image before and after injection of Cariporide.

Changes in the AACID value were visible in the brain of rats after Cariporide injection. The AACID maps obtained after drug injection for one representative animal superimposed on the corresponding anatomical image (Figure 6) show a visible increase in the AACID value twenty minutes after drug injection in the tumor and in the contralateral side followed by a decrease to the baseline levels at 60 min.

**Figure 7:**
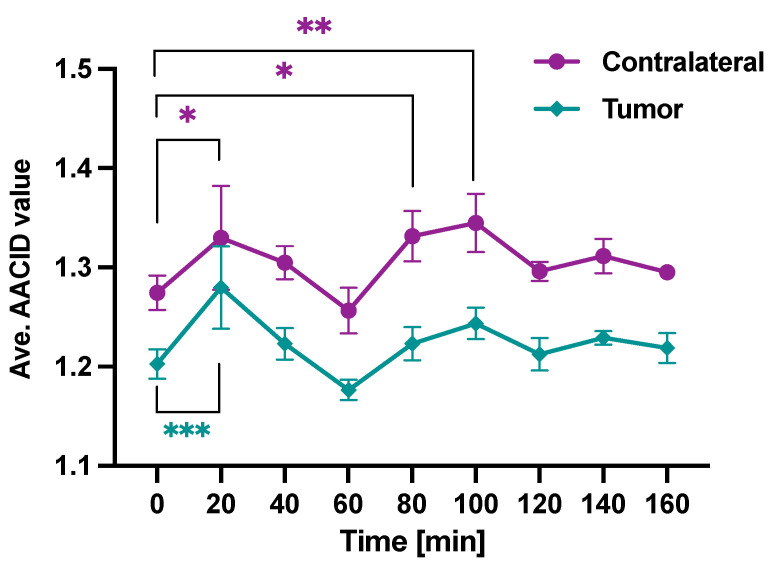
The average AACID value in the tumor and contralateral regions over time before (Time = 0 min) and after (Time = 20–160 min) injection of Cariporide for six rats. Error bars represent the standard error of the mean. The asterisks (* = p<0.05, ** = p<0.01, *** = p<0.001) indicate significant differences in post-hoc Tukey tests.

Figure 7 shows the average AACID value before (time = 0 min) and after (time = 20–160 min) injection of Cariporide (N = 6). Two-way ANOVA showed a difference between contralateral and tumor, as expected, and a trend toward changes over time (p∼0.06). Post-hoc Tukey test was used to compare AACID values at each time point to the baseline. It showed significantly elevated AACID values at 20 min (p<0.05), 80 min (p<0.05), and 100 min (p<0.01) in contralateral tissue, as well as at 20 min in tumor (p<0.0001). The overall response of pH appeared to show an initial increase at 20 min followed by a return to baseline levels, a subsequent increase at 80 min post-injection, followed by another return to baseline levels.

The average AACID value in tumor 20 min post-injection of the drug was ∼6.4% higher than pre-injection, corresponding to a 0.31 ± 0.20 lower pH_*i*_. However, at the second maximum (100 min post-injection of the drug), the AACID value in the tumor was not significantly increased. At this time, the average AACID value in the tumor post-injection was only ∼3.4% higher than pre-injection, corresponding to a 0.16 ± 0.09 lower pH_*i*_. The average AACID value in contralateral tissue increased similarly; however, the immediate effect (at 20 min) after Cariporide injection was less pronounced than the tumor. The average AACID value in the contralateral tissue 20 min post-injection of the drug was ∼4.3% higher compared to pre-injection, corresponding to a 0.22 ± 0.19 lower pH_*i*_. The average AACID value in the contralateral tissue 100 min post-injection of the drug was ∼5.5% higher compared to pre-injection, corresponding to a 0.28 ± 0.15 lower pH_*i*_.

**Figure 8:**
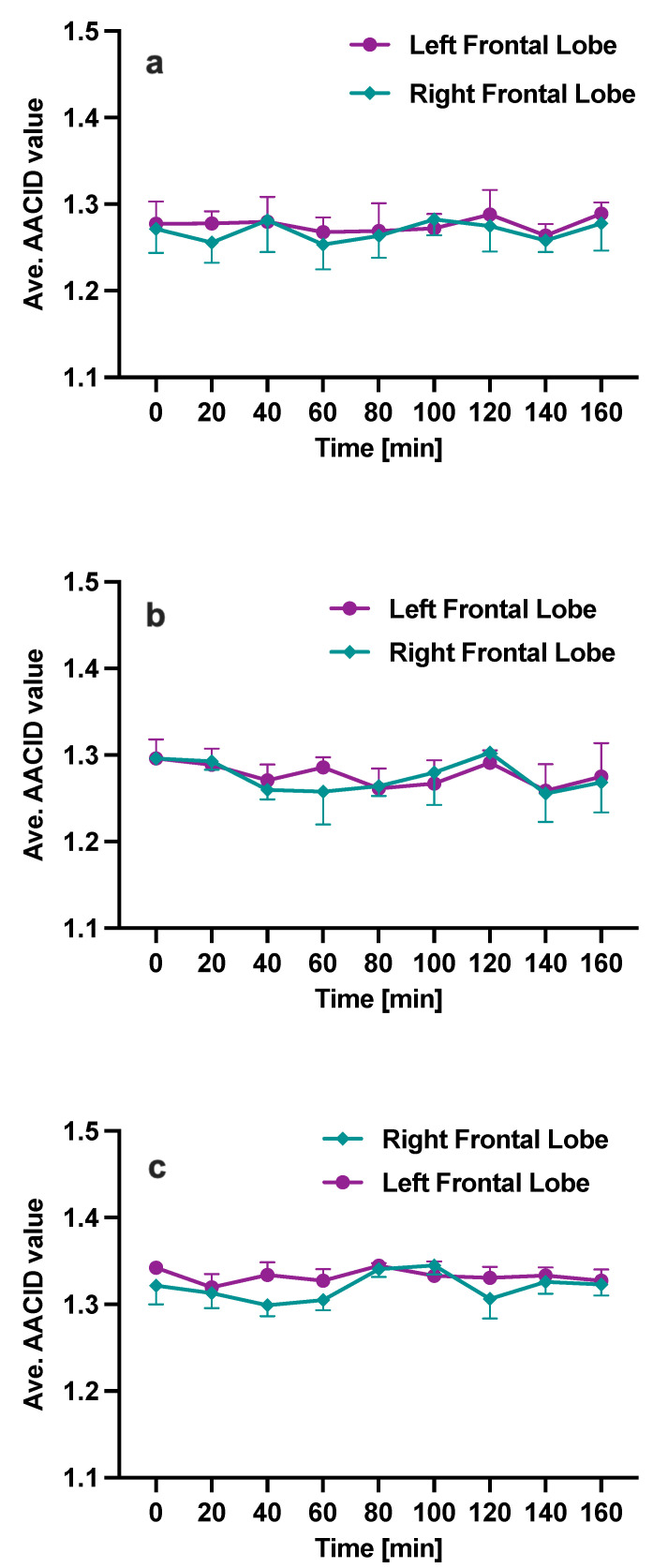
The average AACID value in left and right frontal lobes in control animals before (Time = 0 min) and after (Time = 20–160 min) injection of (**a**) Cariporide dissolved in 100 μL DMSO (N = 4), (**b**) Cariporide dissolved in 10 μL DMSO (N = 3), and (**c**) 100 μL DMSO diluted in 1900 μL PBS (N = 2). The error bars denote the standard error of the mean.

In control animals, no statistical difference in the measured AACID values was observed before (time = 0 min) and after (time = 20–160 min) administration of Cariporide dissolved in 100 μL DMSO (Figure 8a, N = 4) or 10 μL DMSO (Figure 8b, N = 3). Similarly, Figure 8c (N = 2) shows that following only vehicle injection (PBS + 100 μL DMSO), no major changes were observed qualitatively between AACID values measured before (time = 0 min) and after (time = 20–160 min) injection in the right and left frontal lobes of control animals. The data for group 5 are provided for reference only, as no statistical comparisons were made with other groups.

## 4. Discussion

Using AACID-CEST-MRI, the rat C6 glioma model was assessed on day 7–8 and day 14–15 post-implantation, and for the first time, for up to 160 min following a single dose of Cariporide. The observed changes in amide and amine signal ratio (AACID value) were attributed to pH_*i*_ changes. Tumor intracellular pH was found to be basic relative to contralateral tissue at 7–8 days and 14–15 days post-implantation and relative to baseline at 14–15 days post-implantation. Interestingly, the pH in contralateral tissue was lower than baseline at both time points. AACID values in peritumoral regions were intermediate between contralateral tissue and tumor on day 14–15 post-implantation. Finally, a decrease in intracellular pH was observed both within the tumor and in contralateral tissue 20 min following the injection of Cariporide, with a more pronounced effect in tumor tissue. This initial change was followed by a decrease back to baseline and a subsequent increase again in contralateral tissue 80–100 min post-drug injection. No changes in intracellular pH were observed in healthy control animals injected with Cariporide or vehicle.

The current study used the ratiometric AACID-CEST-MRI method as an in vivo indicator of changes in tissue pH_*i*_. This imaging technique provides high-resolution tissue pH-sensitive measurements on a timescale relevant to studying acute pharmacologic effects. The AACID-CEST-MRI method provides a tool sensitive to pH variations in a physiologically relevant range to evaluate the efficacy of pH modulation treatment strategies. While pH modulation has been proposed as a potential treatment for cancer [48,59], evaluation of various approaches must be made in vivo under varying conditions and with different tumor models. Rat glioma models have some advantages over immune-compromised mouse glioma models. The histopathological characteristics of the C6 model, including the presence of pseudopalisade cells, necrotic areas, and micro-vascular proliferation, are similar to those found in GBM patients [60]. The C6 model was also reliable and reproducible, with a very high success rate of intracranial tumor growth and little extracranial growth extension [61], making this model a useful tool for studying and developing new therapies [61,62].

The observation of a basic intracellular pH within the C6 tumor 7–8 days and 14–15 days post-implantation compared to contralateral tissue is consistent with previous works by McVicar et al. [49], Marathe et al. [50], Albatani et al. [24,51,63], in NU/NU mice with the U87MG brain tumor model and Lim et al. [64] in rats with the C6 brain tumor model measured by AACID-CEST-MRI. The current results also agree with a study by Harris et al. that performed amine-CEST-MRI in C57BL/6 mice injected with PBS (control animals) or glioma cells [65]. Their results showed a significant pH difference between tumor and contralateral tissue 14 days post-tumor implantation, which was not observed in the control animals. Hematoxylin and eosin (H&E) staining confirmed that the acidic areas identified by amine CEST were composed of relatively hypercellular and highly necrotic tumor tissue [64,65].

In the current study, lower pH_*i*_ in contralateral tissue was observed on days 7–8 and 14–15 post-implantation relatives to baseline. Either inflammation or reduced blood flow could explain this result. Infiltration and activation of inflammatory cells in the brain may lead to elevation of glucose consumption via the glycolysis pathway, which eventually increases lactic acid. Consequently, pH may decrease [66,67]. Alternatively, as the tumor grows, it may compress surrounding tissue, reducing blood flow and consequently lowering pH_*i*_ [68].

Intermediate values of pH_*i*_ were observed in peritumoral regions. The peritumoral pH may indicate the level of tumor activity or aggression [69]. Cancer cells may invade neighboring tissue, causing neuronal death via glutamate excitotoxicity and degradation of the extracellular matrix via metalloproteinases and other proteases. The inclination of cancer cells to destroy adjacent normal cells causes further invasion and tumor growth, resulting in tissue remodeling and metastases [70]. Tumor metabolism can alter the peritumoral pH microenvironment. The high concentration of protons produced by highly active tumor cells could lead to lower extracellular pH in the peritumoral tissue. Observing a more basic intracellular peritumoral region is consistent with Estrella et al. [69], who monitored tumor invasion and peritumoral pH in severe combined immunodeficiency (SCID) mice-bearing cells derived from human breast cancer and human colon cancer cell lines over time using intravital microscopy. In both cases, a decrease in pH_*e*_ in the tumor and neighboring environment was observed 7 days after implantation. By day 14, acidic regions were recognizable, specifically in the regions the tumors had grown. Histology showed that the tumor edge has an increased expression of NHE1 and GLUT1 (an integral membrane hydrophobic protein that transports glucose, galactose, and glucosamine) compared with the core [69].

NHE1 is the dominant isoform of NHE used by cells to regulate their cell volume and pH_*i*_. The NHE enzyme maintains the acidic tumor microenvironment by reabsorbing extracellular Na^+^ and extruding intracellular H^+^ with a 1:1 stoichiometry. Ma et al. [71] and Rofstad et al. [72] showed that both acidic pH_*e*_ and NHE1 activity elevates tumor invasiveness and are engaged in the resistance of malignant cells to anticancer drugs. Blocking one or some of these regulatory mechanisms has been proposed as an approach to cancer therapy. Decreased tumor pH_*i*_ after injection of a single dose of lonidamine [49], topiramate [50], dichloroacetate [51], and Cariporide [24] has been previously demonstrated using CEST-MRI in a mouse U87MG tumor model. Another study showed that combining five drugs with glucose to target multiple pH regulatory mechanisms also resulted in tumor intracellular acidification in a mouse model of glioblastoma [63]. Additionally, promising results have been obtained in several mouse models of cancer following treatment with proton pump inhibitors [48,73,74]. Blocking NHE reduces the cellular efflux of the H^+^ ions and the cellular influx of Na^+^ ions, consequently decreasing pH_*i*_. Cariporide blocks NHE1 by competing with the Na^+^ ion for its binding site. Cariporide is nontoxic in mammalian cells [59]; therefore, it can be used to promote the action of some chemotherapeutic agents by disrupting the intracellular/extracellular pH gradient. Cariporide has been clinically tested for treating cardiac disease and ischemia-reperfusion injury but never in cancer. A previous study evaluating the chemosensitizing effects of Cariporide in human malignant mesothelioma H-2452 cells pre-adapted with lactic acid showed that Cariporide suppressed growth and promoted apoptosis [23].

The current study is the first to examine the AACID-CEST-MRI (9.4T) indicator of intracellular pH in the rat C6 glioma model as a function of time following a single dose of Cariporide injection. A previous CEST study performed in a NU/NU mouse U87MG glioma model found a significant increase in tumor AACID value with no significant change in AACID value on the contralateral side after Cariporide injection. However, the current study found intracellular acidification in the tumor and the contralateral tissues 20 min following Cariporide injection. Furthermore, acidification was only observed in contralateral tissue at later time points (80–100 min post-injection). Therefore, tumor acidification was not observed to the same extent as hypothesized. Cariporide dissolved in different concentrations of DMSO caused no significant changes in AACID values in healthy brain tissue, in agreement with the study by Albatany et al. [24]. There are several potential explanations for the apparent discrepancy in observed tumor acidification compared to previous studies in the U87MG model. First, the timing of measurements following injection was different in the current study compared to the previous studies. Second, histologic analyses of rat C6 gliomas typically exhibit highly necrotic tumoral tissue [64,65], which is not observed in the NU/NU mouse U87MG glioma model [24]. The timing of measurements and tumor composition may have reduced the observed effect of Cariporide within tumors. Finally, the bulk effect of the tumor on day 14–15 may have impacted contralateral tissue pH. In fact, this is supported by the higher AACID value (lower pH_*i*_) in contralateral tissue on days 7–8 and 14–15 compared to baseline. In addition, the effect of Cariporide in both tumor and contralateral tissues may be influenced by the microvasculature and the ability of Cariporide to infiltrate the tissue or tumor.

Several limitations should be considered when interpreting the results of the current study. First, the sample size (N = 6) used to assess the effect of Cariporide was small. Although sufficiently powered to detect pH_*i*_ changes following drug injection, the study was not designed to examine subtle differences in response between different tissue types. Second, the measured CEST-MRI signal represents contributions from both the intracellular and extracellular compartments; however, it is predominantly weighted to the intracellular space in healthy tissue [56]. The weighting of intracellular and extracellular signals may differ in healthy, compressed, and tumor tissue. Third, the current study used a relatively high dose of Cariporide, 6 mg/kg, to maximize effects. Future studies should examine lower doses, drug combinations, and the addition of glucose in the C6 glioma model. Fourth, tumor-baring rats did not receive the vehicle injection. It is possible that DMSO contributed to observed acidification. A previous study has shown a small contribution of DMSO to changes in the AACID value within the tumor but not in the contralateral tissue [24]. Fifth, Cariporide injection was only performed on day 14–15 when the tumor had become very large. Future studies should examine the effects of Cariporide earlier (during tumor development), which may show greater differential effects compared to contralateral tissue. Finally, B1 magnetic field correction should be considered in future studies to reduce signal variation in CEST images [75,76].

## 5. Conclusions

This study assessed and monitored the effects on tissue pH_*i*_ of Na^+^/H^+^ exchange inhibition with Cariporide in a rat C6 glioma model using AACID-CEST-MRI. As expected, the C6 tumors were found to have higher intracellular pH than healthy brain tissue. Tissue acidification was observed within the first 20 min following the injection of a single dose of Cariporide in both the tumor and contralateral tissue, with a more pronounced effect in tumor tissue. Therefore, pharmacological blockade of the NHE1 protein by Cariporide did modulate intracellular pH, although a differential effect was not observed between tumor and contralateral tissues. In future studies targeting intracellular acidification to improve cancer treatment in humans, Cariporide could be considered as part of the strategy used to modify pH.

## Data Availability

The datasets generated during and/or analyzed during the current study are available from the corresponding author upon reasonable request.

## Abbreviations

The following abbreviations are used in this manuscript:

CNS	Central Nervous System
GBM	Glioblastoma Multiform
AA	Anaplastic Astrocytomas
IDH	Isocitrate Dehydrogenase
KPS	Karnofsky Performance Scale
pH_*i*_	Intracellular pH
pH_*e*_	Extracellular pH
NHE	Na^+^/H^+^ exchangers
NHE1	Na^+^/H^+^ exchanger1
IGF	Insulin-like Growth Factor
EGFR	Epidermal Growth Factor Receptor
GFAP	Glial Fibrillary Acidic Protein
MRI	Magnetic Resonance Imaging
^31^P-MRS	Phosphorus Magnetic Resonance Spectroscopy
CEST	Chemical Exchange Saturation Transfer
WASSR	WAter Saturation Shift Referencing
AACID	Amine and Amide Concentration-Independent Detection
RF	Radio Frequency
APT	Amide Proton Transfer
SNR	Signal-to-Noise Ratio
ROI	Region Of Interest
PBS	phosphate buffered saline
DMSO	Dimethyl Sulfoxide
SQ	Subcutaneous
IP	Intraperitoneal
SCID	Severe Combined Immunodeficiency
